# Diseases of the Nervous System in Old Age

**Published:** 1907-10-26

**Authors:** W. R. Gowers

**Affiliations:** Consulting Physician to University College Hospital.


### Hospital Clinics.

### DISEASES OF THE NERVOUS SYSTEM IN OLD AGE.

By Sir W. R. GOWERS, K.C.B., M.D., F.R.S., Consulting Physician to University College Hospital.

Abstract of a Lecture delivered at the Polyclinic on October 15, 1907.

The subject of the diseases of the nervous system in the aged is one which it is somewhat difficult to make inspiriting or inspiring. A distinction has often been drawn between old age and senility, and they have been defined accordingly in quite different terms. To a certain extent this distinction is a true one, but it must be remembered that decay at some time or other is inevitable; degeneration inevitably begins as age advances. A truer distinction would be one between normal and abnormal senility. The former is the result of that failure of nutrition which in time affects all tissues and organs: the failure of age is a failure of vitality, which comes to an end sooner or later. If this change is local, involving one part or one system more than the rest, we call it a disease, or, in other words, an evidence of abnormal senility. When it attacks all parts simultaneously we call it normal decay.

What is this nutrition which fails as the term of life is reached? It is a change by which new molecules of the tissues are constantly built up from those of the blood plasma under the influence of what we call life. Of life itself we cannot say much more than this, and we shall probably never understand it exactly. All we know is that loss of vitality is a partial failure of these processes of rebuilding.

Loss of vitality is made evident by loss of function long before any defect in structure can be detected; and of the varied functions of the body, first in those of the nervous system, beginning with memory. That process of renewal in certain cells, which is the basis of recollection, is the first to exhibit signs of impairment. As you know, those whose memory fails in old age can often recall the past easily, but not recent events. The longest established changes are those which remain most firmly established and least altered by failure of nutrition, as Ribot has said, "The new perishes, the old endures."

After a time the changes, first seen in loss of function, become visible by the microscope: nutritional change, you must remember, is of necessity colossal before it becomes visible. In the senile brain we see that the nerve cells are less dense; Nissl's bodies become fewer, and inert pigment accumulates in the cells, whose processes become shrunken and short. The fibres, both of the brain and of the spinal cord, become narrowed. Another conspicuous change is increase of neuroglia, the supporting element of the nervous system. Neuroglia at once overgrows whenever there is any wasting process in the nervous system: it is a kind of tissue weed, as well as a supporting structure, and its overgrowth is analogous to that of connective tissue in damaged tissues elsewhere. Like connective tissue, too, such new-formed neuroglia undergoes contraction, and this, with the wasting of the nerve elements, causes a lessening of the bulk of the brain. The convolutions are smaller, and serum occupies the space left vacant. When a case of sudden death in an aged person occurred, the cause was often in olden days said to be "serous apoplexy," a beautiful refuge for those destitute of a better diagnosis.

The vessels are seen on section surrounded by cavities, smooth-walled and of considerable size. It is strange what a very little loss of function this change seems to cause. An apparently normal person has been found after death with a brain in which the perivascular spaces produced a resemblance to a Gruyère cheese. Another kind of space, found especially in the central ganglia, is that which has been termed a lacuna. Lacunation has attracted a good deal of attention in France during the last few years, especially from the French physician Pierre Marie and his pupils. The condition is occasionally seen on the surface of the brain, to which it gives a worm-eaten appearance. Transient hemiplegia, slight mental changes, and other conditions in which there are no definite focal lesions, have all been put down to lacunation. The lacunæ are supposed to be due to degeneration round the smallest arteries, especially those affected with the changes termed "arterio-sclerosis." How far this is correct is somewhat doubtful. Those who have been working at lacunation have ignored the degeneration in the nerve elements themselves, and the effect of other important changes in the vessels, such as atheroma. In some cases the middle cerebral artery is so filled up as barely to admit a pin.

Focal lesions, you may remember, I dealt with here last spring, but there are also many other senile conditions which are of much importance. Some of these are dependent on degenerative changes in other systems, which affect the nervous system indirectly. The organs of circulation, assimilation, and excretion also suffer from loss of vitality, and defects in any of them tell heavily upon the nervous system. The result of such defects is altered composition of the blood: anæmia sometimes, more commonly chemical changes occur which seem to render the blood more or less toxic. In old age this produces a condition analogous to the gouty state: most old people acquire what you might call pseudo-gout. You know how the aged suffer from many rheumatoid manifestations. I believe this is due to a pseudo-gouty state resulting from senile imperfections in the organs. This state promotes growth of the fibrous tissues of the body. Thus Dupuytren's contraction in the palmar fascia is an almost certain indication of a gouty habit. It is important to remember that there are similar effects in the pseudo-gouty state of the old. Other toxæmic conditions also occur in old age, of which we do not know the precise characters. In general one must be on guard against ascribing everything uncertain to a toxæmia, but sometimes we are on safe ground.

I shall leave on one side, though they are most important, the mental states found among the aged, for Dr. Savage has recently described them to you. One characteristic of old persons is their peculiar liability to pain. Thus neuralgia of the fifth cranial nerve is especially common among them, to whom it may make life unendurable: it can, however, be cured by removal of the Gasserian ganglion. Post-herpetic neuralgia, again, is particularly severe and enduring. After an attack of of herpes zoster, the pain in a younger patient would be well in a week, but a man of sixty or upwards may suffer for years. Sir W. Jenner mentioned a patient—and this was before the days of chloroform, mind—who had a piece of his leg removed in the hope of relief from post-herpetic misery. The operation did no good, and the patient shot himself. It may be the greater development of fibrous tissue in the cicatrisation of the inflamed posterior nerve ganglia which causes this pain to be so intense; in some way it must be imperfect recovery owing to defective nutrition.

Sciatica is very common in old people, and is caused by a peri-neuritis due to the pseudo-gouty senile condition. Cases also occur of a curious symmetrical neuritis suggesting an unknown toxic state of the body, of endogenous causation. True degenerative neuritis is also seen, with loss of knee jerk, blunting of sensation, and muscular weakness. Another condition is that of diffuse sclerosis, in which there is a general increase of connective tissue in the spinal cord. This is sometimes very marked in the lateral and posterior tracts, and gives rise to the symptoms of combined sclerosis.

Senile paraplegia of peculiar characters is seen, in which there is a tendency to stiffness and weakness without any change in the knee jerks; this condition seems to me to resemble partial paralysis agitans without tremor. It is always very difficult to influence by treatment.

A few cases of very chronic inflammation have been found in the aged, generally affecting one or both hemispheres. Very little is known of it, but there is strong reason to associate it with a gouty diathesis. The symptoms are like those of cerebral tumour, but optic neuritis never occurs.

It is not to be forgotten that bony changes in the spine or pelvis are often first indicated by nerve symptoms, owing to irritation of nerve roots passing through the foramina. Thus in rheumatoid arthritis of the spinal column nerve pains may alone be complained of, while examination shows the spine to be as rigid as a bar of steel.

Changes in the lining membrane of the labyrinth are common in the old. Vertigo, tinnitus, and deafness, if of gradual onset, are much more common in the old than in the middle-aged. Connective tissue overgrowth is the main factor of these changes.

Another important class of lesions comprises those cases in which local degeneration is much in advance in one system or one place. Local decay of various tracts or groups of cells may fail even in adolescence, *e.g.*, as in Friedreich's ataxia. I have called such forms abiotrophy.

Similar failure in the old may be much in advance of general decay, and some parts or systems of the brain and cord may be much more affected by degeneration than others. Thus bulbar paralysis and progressive muscular atrophy are to be attributed to local failure of nutrition much more severe than that in other parts of the nervous system. Degenerative epilepsy in the old may be due to similar failure; a true epileptic heredity seems occasionally to predispose to it.

Senile tremor is a malady of the old, and so is paralysis agitans. The latter is essentially a disease of the second half of life, but it generally begins before real old age: commonly at fifty to sixty-five, and sometimes much earlier. No doubt such patients are entering upon the period of decay, and yet they are not truly senile.

We often see constant irregular spasm of the face and lips in aged persons. Apparently this is due to inefficient control of the mechanism which puts in action voluntary movements. This condition is quite distinct from senile chorea, which is usually accompanied by mental disturbance.

### Treatment.

Normal senility, of course, nothing can possibly arrest, and hence it is a favourite field for quacks. Injection of testiculine is one of these quack remedies for senility, and you can at any rate save your patients from that. The next best thing to knowing what will do good is knowing what cannot possibly do it.

Treatment suitable for gouty and fibrous rheumatoid affections is suitable for many of these degenerative maladies. Vascular changes can be ameliorated to some slight extent by treatment of the heart and circulatory system. But our best efforts are preventive, and for these we get the least credit; preserve your patient from ill-health, and he takes it as a matter of course that he should be well.

Against systemic degeneration we cannot hope to do much. Always keep the general nutrition in as good a state as you can, and you may thus prevent local changes from becoming greater than they need be. The treatment of senile epilepsy is the same as in younger patients, while for senile tremor nothing can be done. The same holds good for paralysis agitans, except that one must do one's best to lessen the distress of this condition. Hyoscine, by the mouth, or better subcutaneously, undoubtedly does temporary good in this disease; but the drug is cumulative in its action, and alarming heart symptoms are always likely to attend its regular use. Labioglossal paralysis does not respond to strychnine as it does in younger folks, but I think that drug does a little to check the progress of the paralysis.

Equal senility is as impossible as to find two blades of grass the same. If no two oak or elm leaves are ever exactly alike on the tree, much more are they dissimilar in their decay. Hence equable normal senility is not to be looked for, such as that depicted in an allegory by the professor of anatomy at Harvard College. In that allegory there was a one-hoss shay, all parts of which were built to last exactly a hundred years; at the expiration of that time its occupant was suddenly precipitated on to the road, and a little heap of dust was all that remained of his vehicle. In man such a condition, I need hardly say, is never realised.

